# Validation of quantitative magnetic resonance imaging-based apparent bone volume fraction in peri-articular tibial bone of cadaveric knees

**DOI:** 10.1186/1471-2474-15-143

**Published:** 2014-04-29

**Authors:** Jeffrey B Driban, Mary F Barbe, Mamta Amin, Neil S Kalariya, Ming Zhang, Grace H Lo, Anna M Tassinari, Daniel Harper, Lori Lyn Price, Charles B Eaton, Erika Schneider, Timothy E McAlindon

**Affiliations:** 1 Division of Rheumatology, Tufts Medical Center, 800 Washington Street, Box #406, Boston, MA 02111, USA; 2 Department of Anatomy and Cell Biology, Temple University School of Medicine, 3500 North Broad Street, Philadelphia, PA 19140, USA; 3 Medical Care Line and Research Care Line, Houston Health Services Research and Development (HSR&D) Center of Excellence Michael E. DeBakey VAMC, Houston, TX, USA; 4 Section of Immunology, Allergy, and Rheumatology, Baylor College of Medicine, Houston, TX. 1 Baylor Plaza, BCM-285, Houston, TX 77030, USA; 5 Graduate Program in Bioinformatics, Boston University, 44 Cummington Mall, Boston, MA 02215, USA; 6 The Institute for Clinical Research and Health Policy Studies, Tufts Medical Center, and Tufts Clinical and Translational Science Institute, Tufts University, 800 Washington Street, Box #63, Boston, MA 02111, USA; 7 Center for Primary Care and Prevention, Alpert Medical School of Brown University, 111 Brewster Street, Pawtucket, RI 02860, USA; 8 Imaging Institute, Cleveland Clinic Foundation, 9500 Euclid Avenue L10, Cleveland, OH 44195, USA

**Keywords:** Micro-computed tomography, Validation, Magnetic resonance, Osteoarthritis, Trabecula

## Abstract

**Background:**

In the knee, high-resolution magnetic resonance (MR) imaging has demonstrated that increased apparent bone volume fraction (trabecular bone volume per total volume; BV/TV) in the peri-articular proximal medial tibia is associated with joint space narrowing and the presence of bone marrow lesions. However, despite evidence of construct validity, MR-based apparent BV/TV has not yet been cross-validated in the proximal medial tibia by comparison with a gold standard (e.g., micro-computed tomography [microCT]). In this cadaveric validation study we explored the association between MR-based apparent BV/TV and microCT-based BV/TV in the proximal peri-articular medial tibia.

**Methods:**

Fresh cadaveric whole knee specimens were obtained from individuals 51 to 80 years of age with no knee pathology other than osteoarthritis. Ten knees were collected from five cadavers within 10 hours of death and underwent a 3-Tesla MR exam including a coronal-oblique 3-dimensional fast imaging with steady state precession (3D FISP) sequence within 36 hours of death. The specimens were placed in a 4% paraformaldehyde in phosphate buffer within 58 hours of death. After preservation, a subchondral region from the tibial plateau was collected and underwent microCT imaging with a voxel size of 9 μm x 9 μm x 9 μm. A single reader analyzed the microCT images in a similar volume of interest as selected in the MR measures. A different reader analyzed the MR-based trabecular morphometry using a custom analysis tool. To analyze the MR-based trabecular morphometry, a rectangular region of interest (ROI) was positioned on the 20 central images in the proximal medial tibial subchondral bone. The primary outcome measures were MR-based and microCT-based trabecular BV/TV in the proximal medial tibia.

**Results:**

The MR-based apparent BV/TV was strongly correlated with microCT-based BV/TV (*r* = 0.83, confidence interval = 0.42 to 0.96), despite the MR-based apparent BV/TV being systematically lower than measured using microCT.

**Conclusions:**

MR-based apparent BV/TV in the proximal peri-articular medial tibia has good construct validity and may represent an alternative for CT-based BV/TV.

## Background

Peri-articular bone changes may provide important insights into the role of bone in the development and progression of osteoarthritis (OA) [1,2]. In the knee, high-resolution magnetic resonance (MR) imaging has found that increased apparent bone volume fraction (trabecular bone volume per total volume; BV/TV) in the peri-articular proximal medial tibia is associated with joint space narrowing [1] and the presence of bone marrow lesions in knees [2]. These peri-articular bone changes may represent abnormal bone remodeling or microscopic fractures that manifest as attrition. Furthermore, MR imaging confirmed that increased apparent BV/TV in the peri-articular proximal medial tibia is associated with greater peri-articular bone mineral density within the same region of interest (ROI) [1]. These findings indicate that high-resolution MR imaging can yield estimates of apparent BV/TV in the peri-articular proximal medial tibia with good construct validity.

Despite the extensive research, MR-based apparent BV/TV has not yet been cross-validated in the proximal medial tibia by comparison with a gold standard (e.g., micro-computed tomography [microCT]). Prior cross-validation has found that MR-based trabecular metrics are well correlated with CT-based metrics in other anatomic regions (e.g., distal radius, distal tibia, proximal femur, spine, calcaneus) [3-19]. However, the proximal tibia is commonly affected by lesions that can cause abnormal bone marrow signal intensity on MR imaging (e.g., bone marrow lesions in knees with OA or recent knee trauma). Therefore, researchers need to rely on more conservative thresholds to assess apparent BV/TV in the proximal medial tibia compared with other anatomic locations to avoid misclassifying marrow as trabecular bone. We hypothesized that MR-based apparent BV/TV in the proximal medial tibia will have a good relationship (*r* > 0.70) with microCT-based BV/TV, despite generating systematically lower estimates of BV/TV. To test this hypothesis we conducted a cadaveric study to explore the association between MR-based and microCT-based BV/TV in the proximal peri-articular medial tibia.

## Methods

### Cadaveric specimen

Ten fresh cadaveric whole knee specimens were procured from five donors by the National Disease Research Interchange. Inclusion criteria were that the donor’s age was 51 to 80 years and neither knee had pathology with the exception of OA. Race and gender were not donor criteria. Each specimen was at least 16 cm in length and included at least 8 cm on either side of the tibiofemoral joint. Both knees were collected within 10 hours of death, wrapped in gauze, packed in wet ice or ice packs, and sealed for shipping. Within 36 hours of death, the knees underwent MR imaging. After the MR exam, the knees were repackaged with ice packs and shipped to a laboratory for preservation and preparation for microCT. The knees were placed in 4% paraformaldehyde in phosphate buffer within 58 hours of death and were immersion fixed in that solution for 1 week at 4°C, with three changes of fixative. The Institutional Review Board at Tufts Medical Center and Tufts University Health Sciences Campus declared that this study was not human subject research and therefore informed consent for this study was not collected. The National Disease Research Interchange requires that participating hospitals obtain informed consent for the bodies to be used for research purposes from the patient or family.

### Magnetic resonance imaging

**Figure 1:**
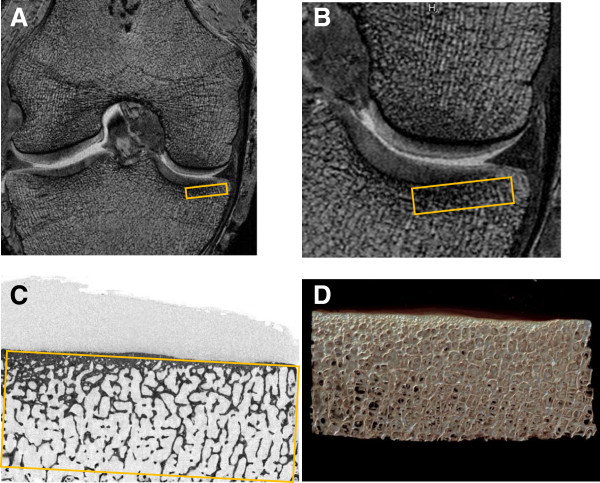
**Trabecular morphometry was measured based on magnetic resonance imaging (MRI; A, B) and micro-computed tomography (micro-CT, C,D). A)** A sample MRI image with the region of interest (ROI) marked in orange. **B)** A zoomed in view of the MRI ROI. **C)** A raw micro-CT image with the ROI indicated in orange. **D)** A reconstructed image based on micro-CT images. All images are from the fourth cadaver’s right knee.

We obtained coronal-oblique 3-dimensional fast imaging with steady state precession (3D FISP) sequences (Figure 1) [17] on a Siemens Trio 3-Tesla MR system and a USA Instruments quadrature transmit-receive knee coil at one of the Osteoarthritis Initiative clinical sites (Memorial Hospital of Rhode Island). The double coronal-oblique orientation was used, with the posterior edge of the medial and lateral femoral condyles in the same slice. Thereafter, the head/foot orientation is aligned parallel to the femoral diaphysis. This orientation has provided reliable assessment of femoro-tibial cartilage loss [20,21] and tibial subchondral trabecular bone [1,2,17] by optimizing the measurement plane to be perpendicular to weight-bearing knee cartilage and subchondral bone. The images were acquired in 10.5 minutes using 72 slices, 1 mm slice thickness, 0.23 mm × 0.23 mm in-plane spatial resolution, 12 cm field of view (FOV), 512 × 512 matrix (interpolated to 1024 × 1024), 4.92 ms echo time (TE) (fat-water in-phase), 20 ms recovery time (TR), 50° flip angle, 180 Hz/pixel readout bandwidth, and phase encode right/left. The chemical shift artifact is 2.4 pixels shifted superior, outside the femoral subchondral bone. We previously used this MR protocol among a convenience sample of the Osteoarthritis Initiative [1,2]. Quality assessments of the MR images were performed for contrast, FOV placement, and absence of motion artifacts.

To determine if there were any bone marrow lesions in the tibia, contiguous sagittal intermediate-weighted turbo spin echo (IW-TSE), fat-suppressed MR images were obtained in the same session as the 3D FISP. Acquisition parameters were: 160 mm FOV, 3 mm slice thickness, 30 ms TE, 3200 ms TR, with an in-plane spatial resolution of 0.357 mm × 0.511 mm, 384 × 269 matrix (interpolated to 512 × 512), “strong” fat suppression (spectral spatial), echo train length 5, 40% phase oversampling, 248 Hz/pixel readout bandwidth, and phase encode anterior/posterior [20].

### Magnetic resonance analyses

A single reader analyzed the coronal 3D FISP images using custom software. As previously described [1,2] a single reader first determined a standardized cortical bone signal intensity threshold by placing 20 regions-of-interest (ROI; 0.69 mm × 0.69 mm) in the cortical bone along the subchondral plate of the medial and lateral femoral condyles (Additional file 1: Figure S1). We used a standardized cortical bone signal intensity as a conservative threshold. We hypothesized that this conservative threshold would cause MR-based apparent BV/TV to systematically underestimate microCT-based BV/TV but also minimize the influence of abnormal bone marrow signal on MR imaging. Additional file 1: Figure S2 is an example of an image after we applied this conservative threshold. To calculate the MR-based trabecular morphometry, a rectangular ROI was positioned on each of the 20 consecutive central MR images [1,2,17] in the proximal medial tibial epiphyseal subchondral bone, just distal to the cartilage (Figure 1). To improve the reliability of the trabecular morphometry metrics, the ROI included the subchondral cortical bone. The ROI had a constant height of 3.75 mm and width of 15.00 mm. Apparent BV/TV was calculated [22] for each image, thereafter the values from the 20 images were averaged. In brief, apparent BV/TV represents the ratio of the number of pixels with signal intensity at or below the signal intensity threshold defined by cortical bone divided by the total [17] number of pixels within the ROI.

Intra-rater (measurement-remeasurement; at least 48 hours apart) reliability was excellent with an intraclass-correlation coefficient (3,1 model) of 0.99 (n = 10) [23]. Furthermore, the smallest and largest paired differences (measurement-remeasurement) in apparent BV/TV were -0.002 and 0.007. The root mean square coefficient of variation (RMS %CV) for the test-retest measurements was 21.4%.

### Micro-computed tomography

The tibial plateau of each knee specimen was divided into medial and lateral compartments using bone saws (Mar-Med Inc., Cleveland, OH, USA). The center of each medial plateau was identified. We then measured 10 mm into the anterior and posterior planes, each, from this center point, and marked the bones with permanent marker at those locations; next we measured 15 mm into the medial planes, each, from this center point, again marking those points before cutting the medial tibia into 14 mm (medial-lateral) × 20 mm (anterior-posterior) × 6 mm in height (with articular cartilage still intact) rectangular pieces using a bone saw (Dremel 4000, Robert Bosch Tools Corporation, Racine WI, USA). These osteochondral specimens were scanned in air with a high resolution, *ex vivo,* cone-beam microCT scanner (Skyscan 1172, 12 megapixel camera model; Microphotonics, Allentown, PA, USA): X-ray source voltage of 59 kV, current of 167 μA, source spot size of 300 nm, aluminum 0.5 mm filter, a rotation step of 0.40°, frame averaging of 4, a ring artifact correction of 10, a beam hardening correction of 40%, 1335 slices, and isotropic voxel resolution of 9 μm. The average scan duration for each osteochondral specimen was 36 minutes. The images were reconstructed into 3D images using cone-beam reconstruction software (Skyscan NRecon, Aartselaar, Belgium) based on the Feldkamp algorithm, a process that yielded 9 μm thick sections in the axial plane. Note, calibration of the Skyscan is performed twice monthly; background corrections were performed before each scan.

### Micro-computed tomography analyses

Structural indices were calculated using the Skyscan CT Analyzer software (CTAn; Aartselaar, Belgium). Trabecular morphometric traits were computed from binarized images using direct 3D techniques that do not rely on prior assumptions from the underlying structures. The volume of interest for trabecular microarchitectural variables was based on the MR ROI, and on the positioning and selection of the osteochondral specimens that underwent the microCT exam. The osteochondral specimens were extracted by identifying the center weight-bearing zone of each medial plateau. We then measured 10 mm into the anterior and posterior planes, each, from this center point – this strategy replicated the 20 consecutive central MR images. Next, we measured 15 mm medial and lateral from this center point. In addition, several study team members (JBD, TEM, GHL, and MFB) reviewed the final microCT volumes to ensure that they corresponded to the MR ROI. The volume of interest was 17 mm (anterior-posterior), 13.729 mm (medial-lateral), and 3.939 mm (vertical). Thresholding or “segmentation” was performed using simple global methods. The binary grayscale range of the Skyscan instrument is from 0 (air, black) to 255 (most solid structure, white), and is indicative of the resorptive properties of the structure scanned, in this case bone. Thus, we used an upper threshold of 255, which captures the densest bone. We also chose a lower threshold of 80 using the grayscale histogram feature of the software, which showed a clear dip in detection of bone versus non-bone structures. We have also used this lower threshold in a number of other publications examining bone structures [24]. The density range of the system is regularly calibrated against "phantoms" of known bone mineral density content and thus Houndsfield units, in which the lower grayscale density of air (0) is equal to -1000 HU, and the highest density of 255 is equal to 9200 Houndsfield units. We computed BV/TV using a marching-cubes algorithm.

### Statistical analyses

The primary outcome measures were MR-based and microCT-based trabecular BV/TV in the proximal medial tibia. We evaluated validity by calculating the association between MR-based and microCT-based trabecular morphometry with Spearman rank correlation coefficients as well as agreement between measures with Bland-Altman analyses. Based on *a priori* power computations, a sample size of 10 knees was expected to provide adequate power to determine criterion validity (*r* > 0.80, power > 0.80, alpha < 0.05). This power calculation is supported by prior research in other anatomical locations, which detected correlation coefficients r > 0.78 using as few as five cadaveric specimens [4]. For these analyses, each knee was considered an independent measurement; however, we also conducted secondary analyses specific to left and right knees.

## Results

**Figure 2:**
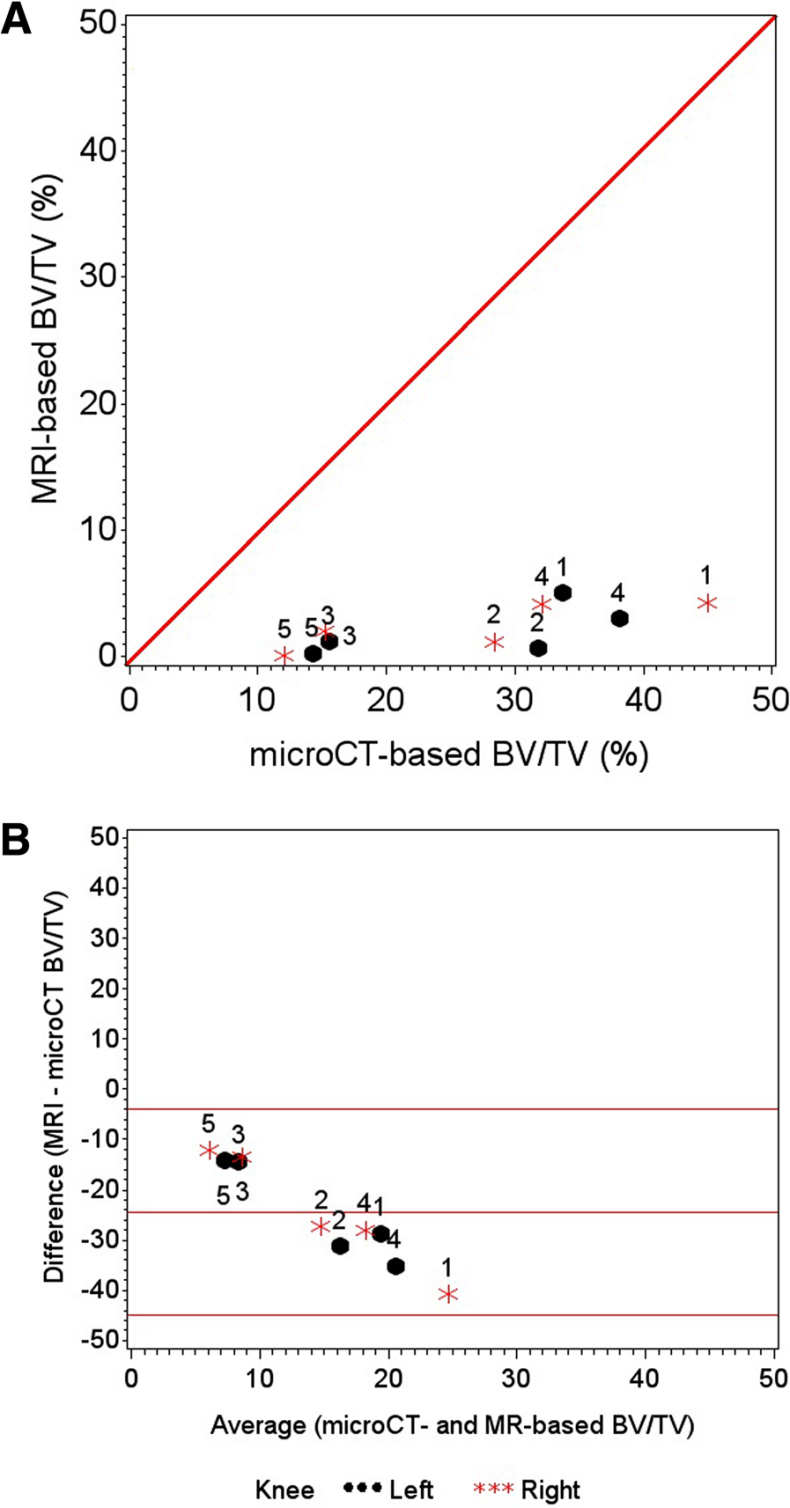
**Plots of magnetic resonance imaging (MRI)-based and micro-computed tomography (CT)-based bone volume fraction (BV/TV).** Scatter **(A)** and Bland-Altman plots **(B)** of MRI-based and microCT-based BV/TV for left (L) and right (R) knees. The red lines in the Bland-Altman plots **(B)** represent the mean difference between MRI and microCT-based BV/TV and the 95% confidence limits of the mean difference.

Table 1 contains the demographic characteristics and quantitative structural data from the five cadavers. None of the knees had an area of high-signal intensity in the tibia on the fat-suppressed IW-TSE images (e.g., bone marrow lesion). In the proximal peri-articular medial tibia, MR-based apparent BV/TV was systematically lower than BV/TV values from microCT images (Figure 2A and 2B). Despite the systematically lower values, the MR-based apparent BV/TV were strongly correlated with microCT-based BV/TV (*r* = 0.83, 95% confidence interval = 0.42 to 0.96; Figure 2A). In secondary analyses, we found similar correlation coefficients among right (r = 0.80, n = 5) and left knees (r = 0.90, n = 5).

**Table 1:** Demographic and structural characteristics of the cadaveric specimens

**Variable**	**Cadaver 1**	**Cadaver 2**	**Cadaver 3**	**Cadaver 4**	**Cadaver 5**
Race	Caucasian	Caucasian	Caucasian	Caucasian	Caucasian
Sex	Male	Male	Female	Male	Female
Age (years)	72	53	57	76	78
Height (m)	1.75	1.70	1.64	1.73	1.68
Weight (kg)	59	70	57	96	48
Right knee: Medial Tibia					
microCT BV/TV (%)	44.98	28.39	15.28	32.16	12.13
MRI BV/TV (%)	4.32	1.23	2.03	4.25	0.09
Left knee: Medial Tibia					
microCT BV/TV (%)	33.74	31.79	15.55	38.11	14.26
MRI BV/TV (%)	5.03	0.62	1.17	3.02	0.20

Notes: microCT = micro-computed tomography, BV/TV = bone volume fraction, MRI = magnetic resonance imaging.

## Discussion

We confirmed that MR-based apparent BV/TV in the proximal peri-articular medial tibia, which has good construct validity [1,2], has good criterion validity when compared to microCT-based BV/TV. Despite MR-based apparent BV/TV being systematically lower than microCT-based BV/TV, MR-based measures may represent an alternative for CT-based BV/TV. MR-based apparent BV/TV may be advantageous for large studies in which the participants are already undergoing MR imaging. Future studies using MR-based apparent BV/TV may help us improve our understanding of the role of subchondral bone changes in osteoarthritis and how bone changes in response to different interventions or exposures.

Prior cross-validation investigations in cadaveric specimens found MR-based trabecular metrics are well correlated with CT-based metrics and have good validity in other anatomic regions (e.g., distal radius, distal tibia, proximal femur, spine, calcaneus) [3-19]. In cadaveric distal radius and tibia specimens, MR-based apparent BV/TV had good correlation (*r* > 0.78; *n* = 5 tibiae, 3 radii) with microCT or high-resolution peripheral computed tomography (HR-pQCT) derived BV/TV [4]. In vivo, the correlation between MR-based apparent BV/TV and HR-pQCT BV/TV was slightly lower than in cadavers in the distal radius (*r* = 0.65 to 0.67, *n* = 5) and slightly better in the distal tibia (r = 0.83; n = 6) [4]. Furthermore, in cadaveric calcaneus specimens (*n* = 40) 3-Tesla MR-based apparent BV/TV had strong correlations (*r* = 0.87) with microCT-based metrics [11]. These results were reinforced by a second study (*n* = 12 cadavers, 24 calcaneus) that reported a high correlation between MR-based apparent BV/TV and basic histomorphometry (*r* = 0.76) [6]. Similarly good correlations have been reported between MR-based apparent BV/TV and multi-slice CT-based measurements of cadaveric femoral heads (r = 0.71, n = 36) [8]. The strong correlation we found in the proximal tibia subchondral bone further supports use of MR-based apparent BV/TV as a robust measurement with good criterion validity in a range of anatomic locations studied to date. In particular, this study supports the use of MR-based apparent BV/TV in the peri-articular proximal medial tibia, which is particularly relevant for knee OA, since the medial tibiofemoral compartment is the compartment most commonly affected [25].

Despite good construct and criterion validity, we found MR-based apparent BV/TV to be systematically lower than microCT-based BV/TV. These differences may be attributed to inherent differences between the imaging modalities, which include the indirect measurement of bone by MR (no signal is observed from bone using clinical MR systems) compared to the direct visualization using microCT, the lower spatial resolution (voxel size: 1000 μm × 230 μm × 230 μm) of MR acquisition compared to that of microCT (voxel size: 9 μm × 9 μm × 9 μm) and thus partial volume averaging on MR possibly reducing the conspicuity of the trabeculae, as well as any post-mortem changes in the MR bone marrow signal influencing the calculation of apparent BV/TV. To minimize post-mortem changes in bone marrow MR-signal characteristics and signal-to-noise after freezing and thawing [26], we did not freeze the cadaver and evaluated the intact knees as quickly as possible (within 36 hrs of death). We also minimized the influence of tissue temperature changes by initiating the MR imaging within 15 minutes of removing a knee from its shipping containers. In addition to these intrinsic differences, we have also used a conservative signal-intensity threshold to define trabeculae. This decision likely introduced a systematic bias that differs from previous reports, which indicated that MR-based apparent BV/TV overestimates BV/TV at other anatomic locations [4,6,11]. The conservative threshold was selected because we believed it to be advantageous in OA, and in the knee in particular, because it should reduce the risk that the presence of MR visible bone marrow abnormalities (e.g., bone marrow lesions) may incorrectly contribute to apparent BV/TV.

While this study provides support for the validity of MR-based apparent BV/TV in the knee, there are several limitations, including the small number of cadavers (5 cadavers, 10 knees) and the very early stage of disease documented by the absence of bone marrow lesions. Although power calculations indicate this number is sufficient to detect criterion validity, it does not eliminate the risk of limiting biologic variability. In specific, our cohort of 10 knees had no MR-visible bone marrow lesions on fat-suppressed IW-TSE images, which are common MR imaging findings in bones of joint affected with OA. Future research is needed to ascertain the optimum threshold as well as to cross-calibrate MR-based apparent BV/TV in the presence of bone marrow lesions among knees with OA [1,2]. Finally, it may be advantageous if future studies determine *in vivo* scan-rescan reproducibility to help estimate sources of measurement error and smallest detectable differences of MR-based BV/TV in the peri-articular proximal medial tibia.

## Conclusions

In conclusion, this study demonstrates that MR-based apparent BV/TV in the proximal medial tibia has good correlation to microCT-based BV/TV. Despite MR-based apparent BV/TV being systematically lower than microCT-based BV/TV, MR-based metrics may represent an alternative for CT-based BV/TV. MR-based apparent BV/TV has good validity and may be advantageous for large studies in which the participants are already undergoing MR imaging.

## Abbreviations

3D FISP: 3-dimensional fast imaging with steady state precession; BV/TV: Bone volume/total volume (bone volume fraction); CT: Computed tomography; FOV: Field of view; IW-TSE: Intermediate-weighted turbo spin echo; HR-pQCT: High-resolution peripheral computed tomography; MR: Magnetic resonance; OA: Osteoarthritis; ROI: Region of interest; RMS %CV: Root mean square coefficient of variation; TE: Echo time; TR: Recovery time.

## Competing interests

The authors have no competing interests that could potentially and inappropriately influence this work.

## Authors’ contributions

JBD participated in the conception and design of the study, acquisition of data (MR imaging data), analyses and interpretation of data, drafting and revising the article, and provided final approval of the version submitted. MFB participated in the conception and design of the study, knee dissections, collection of miocroCT data and analyses and interpretation of data, drafting and revising the article, and provided final approval of the version submitted. Mamta Amin participated in the acquisition of data (microCT data), revising the article, and provided final approval of the version submitted. NSK aided Dr. Barbe in the knee dissections for the acquisition of microCT data. MZ participated in the acquisition of data (developed MR imaging software), revising the article, and provided final approval of the version submitted. GHL participated in the conception and design of the study, interpretation of data, revising the article, and provided final approval of the version submitted. AMT participated in the acquisition of data (developed preliminary MR imaging assessment protocol), revising the article, and provided final approval of the version submitted. DH participated in the acquisition of data (performed MR image assessments), revising the article, and provided final approval of the version submitted. LLP participated in the conception and design of the study, analyses and interpretation of data, revising the article, and provided final approval of the version submitted. CBE participated in the conception and design of the study, analyses and interpretation of data, revising the article, and provided final approval of the version submitted. ES participated in the conception and design of the study, acquisition of data (developed MR imaging sequence), interpretation of data, revising the article, and provided final approval of the version submitted. TEM participated in the conception and design of the study, analyses and interpretation of data, drafting and revising the article, and provided final approval of the version submitted.

## Pre-publication history

The pre-publication history for this paper can be accessed here:

http://www.biomedcentral.com/1471-2474/15/143/prepub

## Supplementary Material

Additional file 1: Figure S1 Examples of the Regions of Interest in the Cortical Bone (yellow dots on femur). We used these regions to determine the cortical bone signal-intensity threshold. The image is from the fourth cadaver’s right knee. **Figure S2:** Binary Image After Applying the Cortical Bone Signal-Intensity Threshold. The image is from the fourth cadaver’s right knee. Click here for file
