# Supplementary material for: Validation of quantitative magnetic resonance imaging-based apparent bone volume fraction in peri-articular tibial bone of cadaveric knees
Source: BMC Musculoskelet Disord. 2014 Apr 29;15:143. doi: 10.1186/1471-2474-15-143 (PMC4021054; doi:10.1186/1471-2474-15-143)

Supplementary Figure 1. Examples of the Regions of Interest in the Cortical Bone (yellow dots on femur). We used these regions to determine the cortical bone signal-intensity threshold. The image is from the fourth cadaver’s right knee.


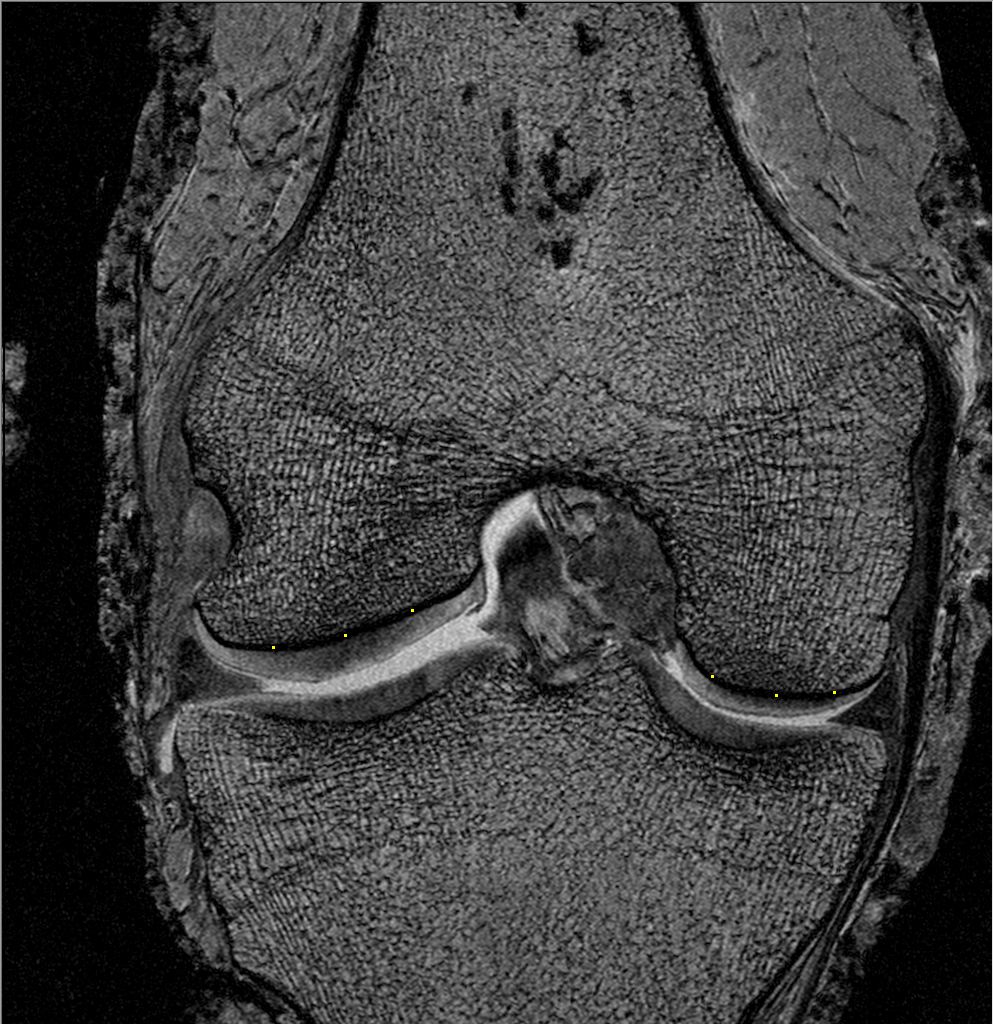


Supplementary Figure 2. Binary Image After Applying the Cortical Bone Signal-Intensity Threshold. The image is from the fourth cadaver’s right knee.


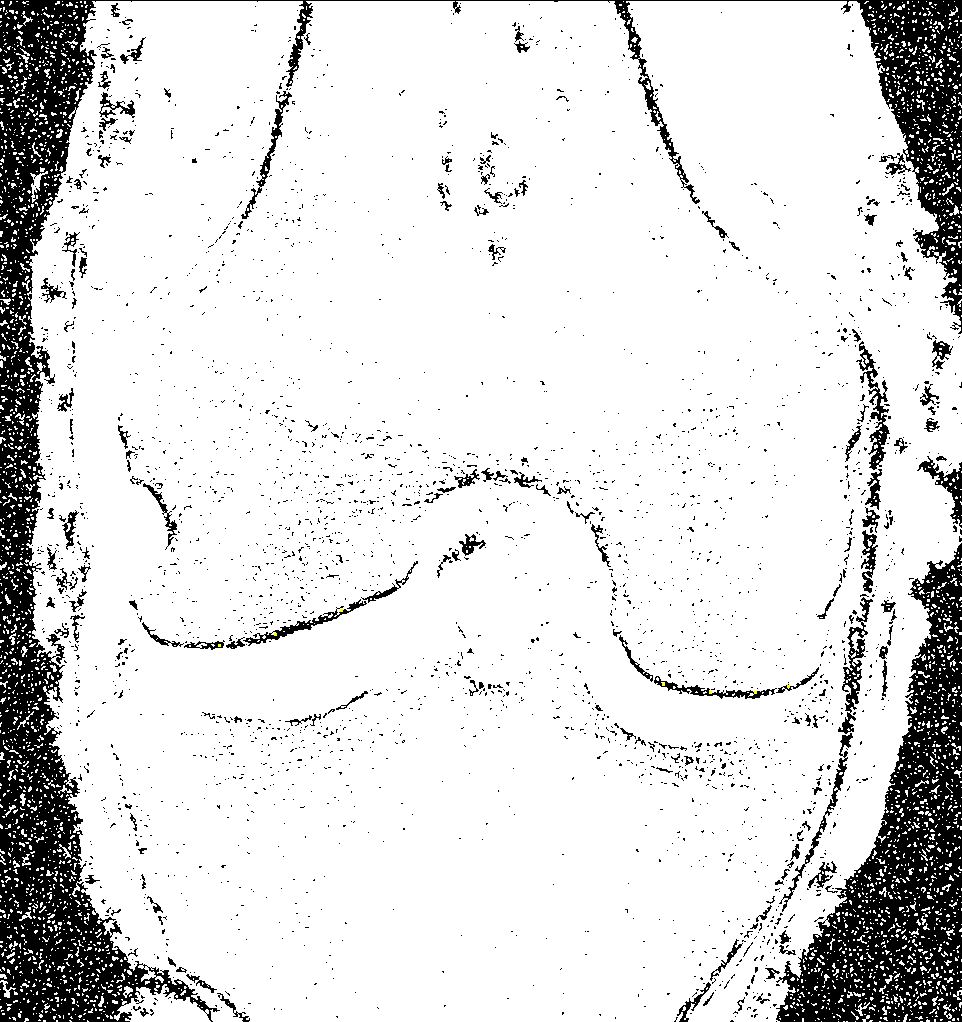

Supplement: Additional file 1: Figure S1 — Examples of the Regions of Interest in the Cortical Bone (yellow dots on femur). We used these regions to determine the cortical bone signal-intensity threshold. The image is from the fourth cadaver’s right knee. Figure S2: Binary Image After Applying the Cortical Bone Signal-Intensity Threshold. The image is from the fourth cadaver’s right knee. [file 1471-2474-15-143-S1.docx]
